# Implementation of a Length Gauge Based on Optical Frequency Domain Reflectometry (OFDR)

**DOI:** 10.3390/s26020393

**Published:** 2026-01-07

**Authors:** Aleksey Shestakov, Dmitriy Kambur, Yuri Konstantinov, Maxim Belokrylov, D. Claude, Igor Shardakov, Artem Turov

**Affiliations:** 1 Institute of Continuous Media Mechanics of the Ural Branch of the Russian Academy of Sciences, Academician Korolev St. 1, Perm 614013, Russia; shap@icmm.ru (A.S.); kambur.dima@gmail.com (D.K.); belokrylovme@gmail.com (M.B.); cdf750@yandex.ru (D.C.); shardakov@icmm.ru (I.S.); artemtur442@gmail.com (A.T.); 2 General Physics Department, Applied Mathematics and Mechanics Faculty, Perm National Research Polytechnic University, Komsomolsky Prospect, 29, Perm 614990, Russia

**Keywords:** optical length gauges, optical ranging, optical frequency domain reflectometry, interferometry, optical frequency scanning

## Abstract

Optical frequency domain reflectometry (OFDR) is a widely used method for measuring optical lengths to backscattering points in optical fibers and integrated optical chips. However, its application for measuring absolute distances in other media, including free space, remains insufficiently studied. This work aims to solve two main challenges in developing a free-space distance measurement method based on OFDR. The first one is the adaptation of the standard OFDR method to air-based measurements, considering the complex and/or atypical composition of the optical line, including the combination of fiber and air, as well as differing chromatic dispersion. The second task is the calibration of the reflectometer to ensure high measurement accuracy. The article proposes a mathematical framework for eliminating the influence of chromatic dispersion, based on signal transformation and the introduction of an equivalent phase of the reference interferometer. The method was verified experimentally. The experimental setup included an OFDR system, a collimator, and a corner reflector movable along a 2-m rail. An important result is the development and testing of a dispersion compensation method, which eliminated peak broadening in the trace as the distance increased, maintaining its width at a level of tens of microns. Through calibration using an interferometric fringe-counting method, a frequency-to-distance conversion coefficient was determined, ensuring measurement accuracy up to 2 μm. Thus, the study demonstrates the feasibility of adapting OFDR for precise distributed distance measurements in free space and in complex or otherwise non-standard structured environments, significantly expanding the application scope of the technology.

## 1. Introduction

Distributed fiber-optic measurement systems have long been applied to advanced technologies, finding application in telecommunications [1,2,3,4,5], monitoring critical man-made facilities [6,7,8,9,10], geosciences, the oil and gas industry [11,12,13,14,15], and the cable and fiber industry [16,17,18,19,20]. One of the most promising approaches developed in this area is optical frequency domain reflectometry (OFDR). It is a highly sensitive method for measuring the intensity of reflected optical radiation from all points of the analyzed optical line. This method is applicable to the study of fiber-optic cables, optical fibers, and integrated optical chips [21,22,23,24,25,26,27,28]. Due to its unique properties, this method has found widespread application in many fields of science and technology. Among the many distributed sensing technologies, OFDR stands out due to its high spatial resolution and wide dynamic range.

The primary application of frequency domain reflectometry in its early days was the diagnostics of non-standard optical fibers and integrated optical circuits. Due to the method’s high spatial resolution and high dynamic range, such systems allow for the visualization of back-reflections with a high degree of localization and detail [29,30,31,32,33]. As new fibers, fiber assemblies, and integrated circuits continue to be produced and improved, this application of OFDR remains highly relevant [34,35,36,37,38]. Another application of the method is optical sensorics. The sensing applications of OFDR are well described in the following review paper [39]. Specifically, it notes that the OFDR method is actively used to verify mathematical models describing the deformation of composite structures under mechanical loads. For example, aircraft wing deformation is modeled using the finite element method (FEM), and these results are then compared with the results of a full-scale experiment obtained using fiber-optic sensors. This comparison helps confirm the accuracy of the modeling and identify potential problems at the design stage, reducing testing costs and accelerating the development of new products. Studies of this kind are widely presented in modern scientific literature [40,41,42,43,44]. Another unique application of OFDR is thermal monitoring of lithium-ion batteries and electric motors in next-generation vehicles. Distributed temperature detection within the battery or electric drive enables early detection of overheating of individual components and prevents hazardous situations such as battery fires or engine failure.

Another noteworthy application of OFDR is the integrity monitoring of composite pressure vessels (COPVs) [45]. The use of strain gauges integrated directly into composite materials allows for precise monitoring of stress changes and the detection of localized structural damage down to the level of microscopic defects. Such gauges help prevent catastrophic failures by early detection of potentially hazardous areas and assessing the residual strength of the structure. Such research is also widely represented in the scientific literature [46,47,48,49,50].

Thus, OFDR technology is a powerful tool for studying back-reflections in optical fibers and other media. It is used for monitoring and diagnosing complex engineering systems, thereby increasing their reliability. This technology integrates precise strain and temperature measurements into a single design and operation process, significantly reducing testing costs and extending the service life of critical components.

For absolute distance measurement in free space, several high-accuracy techniques exist, such as laser interferometry, frequency-modulated continuous-wave (FMCW) LiDAR, or phase-shift rangefinders. These methods, however, are inherently designed for point-to-point ranging. In contrast, OFDR provides a distributed response, capable of resolving multiple reflection points along an optical path. This makes it uniquely suited for applications where the integrity of the entire optical line must be monitored—for instance, in complex experimental setups featuring sequential elements with different optical properties (e.g., fiber segments, air gaps, and various optical components). Adapting OFDR to such heterogeneous environments, particularly those containing free-space sections, remains an unresolved challenge, which this work addresses.

However, the scientific literature currently lacks references to the use of optical frequency domain reflectometry for length measurements in open space. This need arises in a range of applications, as well as in monitoring experimental optical circuits constructed from elements with widely varying optical properties, including those with air sections. It is expected that the approach successfully applied in waveguide structures can also be successfully applied in open environments or lines containing both fiber and air sections, where measurements are performed using collimated radiation reflected from a special surface and returned to an optical frequency-domain reflectometer for subsequent analysis. Adapting the OFDR method to the air environment will not only allow for the localization of the reflector with high spatial accuracy, obtaining the absolute distance value, but also for studying certain features of the propagation medium, as well as surface parameters.

This study is the first step in developing free-space measurements using optical frequency domain reflectometry. It addresses two objectives:

1. Adapting the standard optical frequency domain reflectometry method to free-space length measurements. Typically, standard optical frequency domain reflectometers consist of two interferometers: a primary interferometer and a reference interferometer. In standard configurations, the arms of both interferometers are made of the same material—germanium oxide-doped quartz glass. Therefore, both materials have comparable optical parameters, including chromatic dispersion. When switching to air measurements, one of the primary interferometer arms becomes a composite interferometer: the part inside the system remains based on optical fiber, while the second part, representing the length being measured, becomes air-based. If measurements are performed in standard mode, spatial resolution will significantly degrade with increasing measurement range. This is due to an increase in the width of the trace peak corresponding to the reflector. To address this drawback, a mathematical framework is proposed and described below;
2. Reflectometer calibration, allowing for length measurements with high accuracy. Typically, the optical lengths of the interferometer arms included in a reflectometer are not known with high accuracy. Therefore, a calibration method based on comparing reflectometer readings with a well-known interferometric method for measuring displacements is proposed in this paper.

## 2. Experimental Setup

**Figure 1:**
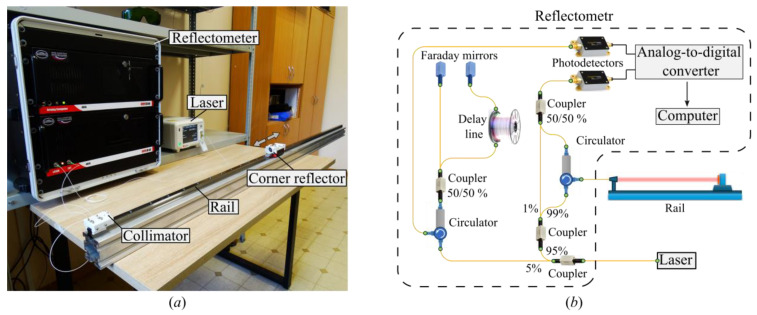
Experimental setup, (**a**) photo, (**b**) optical scheme.

A photograph of the experimental setup is shown in Figure 1a. The setup consists of a reflectometer, a laser source, and a variable-length overhead optical line. This line is formed by a metal rail mounted on a laboratory table using vibration-damping pads, a fiber collimator, and a corner reflector. The rail is 2 m long. The collimator is positioned so that the optical radiation emitted from it is directed toward the reflector and propagates parallel to the rail axis. The corner reflector is mounted on a carriage, allowing it to move along the rail.

The fiber collimator provided a beam with an output diameter of 1.0 mm. Over the maximum measurement distance of 1.8 m, the beam divergence resulted in a spot diameter of less than 5 mm, ensuring it remained well within the effective aperture of the 25 mm corner-cube retroreflector used. To avoid parasitic reflections from the front surface of the retroreflector, it was slightly tilted relative to the optical axis, ensuring that only the triple reflection from the cube’s internal facets contributed to the main signal peak. The carriage was moved manually along the rail without a mechanical drive. All experiments were conducted under monitored laboratory conditions: the air temperature was 24.3 °C with variations within ±0.1 °C, atmospheric pressure was 742 mmHg, and relative humidity was 52%. These parameters remained stable during the measurements.

The ARFA-001a optical frequency domain reflectometer (ORMS Lab, LLC, Perm, Russian Federation) was used in the study. It was previously developed by laboratory staff [51]. The reflectometer’s optical scheme is shown in Figure 1b. According to this scheme, the optical radiation generated by the laser source is divided into two parts using a fiber splitter. These parts are directed to the reference and main interferometers. Both interferometers include delay lines. In the reference interferometer, the delay line is a buffer fiber, which is located inside the device at rest, insulated from mechanical and thermal influences. In the main interferometer, the delay is realized by the line under study. In this implementation of the scheme, the main interferometer is implemented using the Mach-Zehnder principle, and the reference interferometer is a Michelson interferometer. Both interferometers are implemented using fiber splitters and three-port fiber circulators. A list of all components used in the setup is presented in Table 1. A detailed description of the reflectometer’s operation is given in the following papers [52,53,54].

The laser frequency sweep time was 1.5 s. The photodetector signals were sampled at 25 MS/s using a 16-bit ADC.

## 3. The Method for Eliminating Chromatic Dispersion

**Figure 2:**
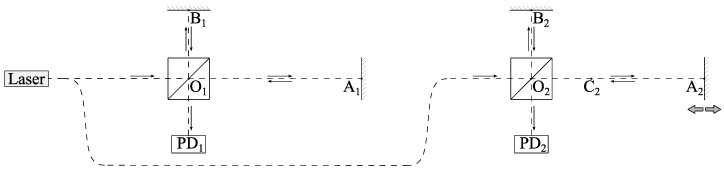
Simplified interpretation of the main and auxiliary interferometers; PD—photodetector, A, B—reflectors, O—splitter, C—specific point. The subscripts ‘1’ and ‘2’ denote quantities related to the reference and main interferometers, respectively.

Figure 2 shows a simplified schematic of the reflectometer. It consists of two interferometers. The first interferometer (with points O_1_, A_1_, B_1_) is considered as the reference one, and the second (with points O_2_, A_2_, B_2_, C_2_) as the primary one. Subscripts ‘1’ and ‘2’ denote the corresponding interferometers throughout the following theoretical analysis. In some general derivations applicable to any interferometer, these subscripts may be omitted for brevity, which will be explicitly stated.

For each interferometer, at point O, the laser beam emitted by the source is split into two. The first one is directed to reflector A, the other one travels to reflector B. After reflection, the beams return to point O, from which the combined beam is directed to the photodetector.

This paper considers two configurations of the primary interferometer. In the first, it is considered homogeneous, while in the second, arm OA is divided into two sections, OC and CA, whose refractive indices are different. The reference interferometer is considered homogeneous in both cases.

When the primary and reference interferometers are homogeneous and made of the same type of optical fiber, this does not present any additional complications, as the chromatic dispersion functions for these media are similar. However, when the reference interferometer is made of fiber and the primary interferometer is made of a different type of fiber, or when its optical medium is air, the trace peak corresponding to the reflector begins to broaden with increasing spatial coordinates. The situation becomes even more complex when the primary interferometer is inhomogeneous, with one part made of fiber and the other consisting of air. All these cases are discussed below.

### 3.1. Reflectometer with Homogeneous Interferometers

Let’s consider the signal received by the photodetectors of homogeneous interferometers. The form of this function will be the same for both interferometers. Here, subscripts are omitted for the sake of generality.

It is well known that interference beats recorded by an interferometer’s photodetector are described by a cosine function, the argument of which is the product of the circular frequency of the optical radiation and the difference in the light transit times along the interferometer’s arms [55,56].

(1) $$I_{PD}=\cos\left(\omega \times ((T_{OA}-T_{OB})\right),$$

where I_PD_ is the intensity of the optical signal recorded by the photodetector, ω is the circular frequency of optical radiation, and T_OA_ and T_OB_ are the transit times of the corresponding paths in the interferometer. These time delays can be expressed in terms of the arm lengths and the refractive index of the medium as follows:

(2) $$\begin{array}{l} T_{OA}=\frac{2\times L_{OA}\times n\left(\omega \right)}{c_{0}},\\ T_{OB}=\frac{2\times L_{OB}\times n\left(\omega \right)}{c_{0}}, \end{array}$$

where L_OA_ and L_OB_ are the lengths of the interferometer arms; n(ω) is the refractive index of a medium, which is a function of frequency; c_0_ is the speed of light in vacuum. Substituting (2) into (1):

(3) $$I_{PD}=\cos\left(2\times \omega \times \frac{n\left(\omega \right)}{c_{0}}\left(L_{OA}-L_{OB}\right)\right)=\cos\left(2\times \omega \times n\left(\omega \right)\times \frac{\Delta L}{c_{0}}\right),$$

where ΔL denotes the difference in the lengths of the interferometer arms. This relationship is valid for any homogeneous interferometer.

Considering the photodetector signal of the reference interferometer, one can use the following notation: ω = ω(t) is the monotonic function, at time t = 0, the optical radiation frequency begins to sweep, and at t = t_end_, the sweeping is completed. Then, the signal on the photodetector of the first interferometer will have the form (4).

(4) $$I_{PD1}(t)=\cos\left(2\times \omega \left(t\right)\times n_{1}\left(\omega \left(t\right)\right)\times \frac{\Delta L_{1}}{c_{0}}\right).$$

Using the Hilbert transform, one can determine the phase of the I_PD1_(t) function oscillations. Then the phase unwrapping procedure over the interval $t\in \left[0,t_{end}\right]$ is performed. The phase value at t = 0 is defined as an integer number of periods, so the total phase of the harmonic function (4) can be expressed as follows:

(5) $$2\times \omega \left(t\right)\times n_{1}\left(\omega \left(t\right)\right)\times \frac{\Delta L_{1}}{c_{0}}=\varphi_{1}\left(t\right)+2\pi \times k_{1},$$

where φ_1_(t) is the unfolded phase of the signal I_PD1_(t); k_1_ is an integer that determines the number of periods at t = 0. From Equation (5), one can express the circular frequency of optical radiation ω(t):

(6) $$\omega \left(t\right)=\frac{1}{2}\times \frac{1}{n_{1}\left(\omega \left(t\right)\right)}\times \frac{c_{0}}{\Delta L_{1}}\times \left(\varphi_{1}\left(t\right)+2\pi \times k_{1}\right).$$

Considering the second interferometer:

(7) $$I_{PD2}(t)=\cos\left(2\times \omega \left(t\right)\times n_{2}\left(\omega \left(t\right)\right)\times \frac{\Delta L_{2}}{c_{0}}\right).$$

According to Figure 2, the optical radiation frequency is the same for the first and second interferometers. Based on this, one can substitute the frequency expression (6) into relation (7):

(8) $$I_{PD2}\left(t\right)=\cos\left(\frac{n_{2}\left(\omega \left(t\right)\right)}{n_{1}\left(\omega \left(t\right)\right)}\times \frac{\Delta L_{2}}{\Delta L_{1}}\times \left(\varphi_{1}\left(t\right)+2\pi \times k_{1}\right)\right).$$

In the case that φ_1_(t) is monotonic, one can construct the inverse function t(φ_1_). Using this dependence, a change in variables can be made. The signal of the second interferometer I_PD2_(t) will be expressed as a function of the phase φ_1_ of the first one:

(9) $$I_{PD2}\left(t\right)=I_{PD2}\left(t\left(\varphi_{1}\right)\right)=I_{PD2}\left(\varphi_{1}\right).$$

This will eliminate time from subsequent relationships. Finally, the resulting trace will be independent of the speed and uniformity of the optical radiation frequency sweeping. The circular frequency of the optical radiation can be expressed in a similar manner:

(10) $$\omega \left(t\right)=\omega \left(t\left(\varphi_{1}\right)\right)=\omega \left(\varphi_{1}\right).$$

Substituting expressions (9) and (10) into (8):

(11) $$I_{PD2}\left(\varphi_{1}\right)=\cos\left(\frac{n_{2}\left(\omega \left(\varphi_{1}\right)\right)}{n_{1}\left(\omega \left(\varphi_{1}\right)\right)}\times \frac{\Delta L_{2}}{\Delta L_{1}}\times \left(\varphi_{1}+2\pi \times k_{1}\right)\right).$$

From (11), it follows that if the optical media of the first and second interferometers are identical, then the refractive index functions are also identical, and therefore can be canceled. As a result, (11) takes the simple form of:

(12) $$I_{PD2}\left(\varphi_{1}\right)=\cos\left(\frac{\Delta L_{2}}{\Delta L_{1}}\times \left(\varphi_{1}+2\pi \times k_{0}\right)\right).$$

In this case, the angular frequency of the I_PD2_(φ_1_) signal is equal to $\frac{\Delta L_{2}}{\Delta L_{1}}$. The reflector’s amplitude-frequency response exhibits a single narrow peak. When the media are different—for example, the first interferometer is made of quartz fiber, while the second interferometer’s optical medium is air—the refractive indices cannot be omitted, and different frequencies correspond to the same $\frac{\Delta L_{2}}{\Delta L_{1}}$ value. To overcome this problem, (11) can be transformed in the following way:

(13) $$I_{PD2}\left(\varphi_{1}\right)=\cos\left(\frac{\Delta L_{2}}{\Delta L_{1}}\times \frac{n_{2}\left(\omega \left(\varphi_{1}\right)\right)}{n_{1}\left(\omega \left(\varphi_{1}\right)\right)}\times \left(\varphi_{1}+2\pi \times k_{1}\right)\right)=\cos\left(\frac{\Delta L_{2}}{\Delta L_{1}}\times Q\left(\varphi_{1},k_{1}\right)\right).$$

Here, Q(φ_1_, k_1_) is a function of the variable φ1 and the constant k_1_. When sweeping the optical radiation frequency, k_1_ does not change. It depends on the initial sweeping frequency ω(0). To determine the function Q(φ_1_, k_1_), the Hilbert transform is used to determine the phase of the oscillations of the function I_PD2_(φ_1_). Then, the phase unwrapping procedure is performed over the interval $\varphi_{1}\in \left[\varphi_{1}\left(0\right),\varphi_{1}\left(t_{end}\right)\right]$:

(14) $$\varphi_{2}\left(\varphi_{1}\right)+2\pi \times k_{2}=\frac{\Delta L_{2}}{\Delta L_{1}}\times Q\left(\varphi_{1},k_{1}\right).$$

Using the least squares method, φ_2_(φ_1_) can be expanded into a power series:

(15) $$\varphi_{2}\left(\varphi_{1}\right)=p_{0}+p_{1}\times \varphi_{1}+p_{2}\times \varphi_{1}^{2}+p_{3}\times \varphi_{1}^{3}.$$

The function Q(φ_1_, k_1_) can be represented in a similar way:

(16) $$Q\left(\varphi_{1},k_{0}\right)=q_{0}+q_{1}\times \varphi_{1}+q_{2}\times \varphi_{1}^{2}+q_{3}\times \varphi_{1}^{3}.$$

In this expansion, the q coefficients depend on the constant k_1_. Substituting expansions (15) and (16) into (14): way:

(17) $$p_{0}+p_{1}\times \varphi_{1}+p_{2}\times \varphi_{1}^{2}+p_{3}\times \varphi_{1}^{3}+2\pi \times k_{2}=\frac{\Delta L_{2}}{\Delta L_{1}}\times \left(q_{0}+q_{1}\times \varphi_{1}+q_{2}\times \varphi_{1}^{2}+q_{3}\times \varphi_{1}^{3}\right).$$

The left and right sides of (17) contain the signal phase values. The signal’s amplitude-frequency response is independent of the constant phase component, so we will exclude it from (17):

(18) $$p_{1}\times \varphi_{1}+p_{2}\times \varphi_{1}^{2}+p_{3}\times \varphi_{1}^{3}=\frac{\Delta L_{2}}{\Delta L_{1}}\times \left(q_{1}\times \varphi_{1}+q_{2}\times \varphi_{1}^{2}+q_{3}\times \varphi_{1}^{3}\right).$$

As a result of identical transformations, the relation (18) is reduced to the form (19):

(19) $$p_{1}\times \left(1+\frac{p_{2}}{p_{1}}\times \varphi_{1}+\frac{p_{3}}{p_{1}}\times \varphi_{1}^{2}\right)\times \varphi_{1}=\frac{\Delta L_{2}}{\Delta L_{1}}\times q_{1}\times \left(1+\frac{q_{2}}{q_{1}}\times \varphi_{1}+\frac{q_{3}}{q_{1}}\times \varphi_{1}^{2}\right)\times \varphi_{1}.$$

Equating the corresponding coefficients:

(20) $$\frac{\Delta L_{2}}{\Delta L_{1}}\times q_{1}=p_{1},\;\;\;\;{q^{\prime }}_{2}=\frac{q_{2}}{q_{1}}=\frac{p_{2}}{p_{1}},\;\;\;\;{q^{\prime }}_{3}=\frac{q_{3}}{q_{1}}=\frac{p_{3}}{p_{1}}.$$

Taking into account the obtained expansion coefficients, relation (13) can be expressed in the following form:

(21) $$I_{PD2}\left(\varphi_{1}\right)=\cos\left(\frac{\Delta L_{2}}{\Delta L_{1}}\times q_{1}\times \left(1+{q^{\prime }}_{2}\times \varphi_{1}+{q^{\prime }}_{3}\times \varphi_{1}^{2}\right)\times \varphi_{1}\right).$$

To ensure the unambiguity of the signal frequency and the position of the reflector, the equivalent phase function of the reference interferometer is introduced:

(22) $${\tilde{\varphi }}_{1}\left(\varphi_{1}\right)=\left(1+{q^{\prime }}_{2}\times \varphi_{1}+{q^{\prime }}_{3}\times \varphi_{1}^{2}\right)\times \varphi_{1}.$$

Expressing the signal function of the second interferometer as a function of the equivalent phase:

(23) $$I_{PD2}\left(\varphi_{1}\right)=I_{PD2}\left(\varphi_{1}\left({\tilde{\varphi }}_{1}\right)\right)=I_{PD2}\left({\tilde{\varphi }}_{1}\right).$$

With this variable the function will take a simple form of:

(24) $$I_{PD2}\left({\tilde{\varphi }}_{1}\right)=\cos\left(\frac{\Delta L_{2}}{\Delta L_{1}}\times q_{1}\times \left(\left(1+{q^{\prime }}_{2}\times \varphi_{1}+{q^{\prime }}_{3}\times \varphi_{1}^{2}\right)\times \varphi_{1}\right)\right)=\cos\left(\frac{\Delta L_{2}}{\Delta L_{1}}\times q_{1}\times {\tilde{\varphi }}_{1}\right).$$

As a result, on the amplitude-frequency characteristic $I_{PD2}\left({\tilde{\varphi }}_{1}\right)$, one value of $\frac{\Delta L_{2}}{\Delta L_{1}}$ corresponds to one frequency.

As noted above, in a real-world reflectometer optical design, where the measured line has an air section, the main interferometer consists of a quartz and an air section. This case will be discussed below.

### 3.2. Reflectometer with an Inhomogeneous (Piecewise Homogeneous) Main Interferometer

Considering Figure 2 again and assuming the main interferometer to be inhomogeneous this time, while the reference interferometer remains unchanged (homogeneous), one can express the signal from the main interferometer’s photodetector. For clarity, the subscript 2 will be omitted. One should also assume that the OC section is made of germanium-doped quartz glass, and the CA section is made of air. The optical paths of the OC and OB have a refractive index of n_1_(ω), while the CA path has a refractive index of n_2_(ω). In this case, T_OA_ (Equation (2)) is the sum of the delays in the two sections:

(25) $$T_{OA}=T_{OC}+T_{CA}.$$

(26) $$\begin{array}{c} T_{OA}=\frac{2\times L_{OC}\times n_{1}\left(\omega \right)}{c_{0}}+\frac{2\times L_{CA}\times n_{2}\left(\omega \right)}{c_{0}},\\ T_{OB}=\frac{2\times L_{OB}\times n_{1}\left(\omega \right)}{c_{0}}. \end{array}$$

Substituting (26) into the ratio (1):

(27) $$\begin{array}{c} I_{PD2}=\cos\left(2\times \frac{\omega }{c_{0}}\times \left(\left(L_{OC}-L_{OB}\right)\times n_{1}\left(\omega \right)+L_{CA}\times n_{2}\left(\omega \right)\right)\right)\\ =\cos\left(2\times \frac{\omega }{c_{0}}\times \left(\Delta L_{21}\times n_{1}\left(\omega \right)+L_{22}\times n_{2}\left(\omega \right)\right)\right). \end{array}$$

Here, the following notations are additionally introduced: ΔL_21_ = L_OC_ − L_OB_, L_22_ = L_CA_. The reference interferometer is similar to the one considered in the previous section, so relation (6) is also valid in this case. Substituting the circular frequency obtained on the reference interferometer into expression (27):

(28) $$I_{PD2}\left(\varphi_{1}\right)=\cos\left(\left(\frac{\Delta L_{21}}{\Delta L_{1}}+\frac{L_{22}}{\Delta L_{1}}\times \frac{n_{2}\left(\omega \left(\varphi_{1}\right)\right)}{n_{1}\left(\omega \left(\varphi_{1}\right)\right)}\right)\times \left(\varphi_{1}+2\pi \times k_{1}\right)\right).$$

Then, a Fourier transform of signal I_PD2_(φ_1_) should be performed. The amplitude-frequency characteristic exhibits a narrow peak corresponding to point C (Figure 2). At this point, the quartz fiber ends and the optical path then passes through air. The angular frequency of this peak is equal to $\frac{\Delta L_{21}}{\Delta L_{1}}$.

Based on the real signal I_PD2_(φ_1_), using the Hilbert transform the complex signal I^C^_PD2_(φ_1_) can be determined. According to this transform, expression (28) takes the following form:

(29) $$I_{PD2}^{c}\left(\varphi_{1}\right)=\exp\left(j\times \left(\frac{\Delta L_{21}}{\Delta L_{1}}+\frac{L_{22}}{\Delta L_{1}}\times \frac{n_{2}\left(\omega \left(\varphi_{1}\right)\right)}{n_{1}\left(\omega \left(\varphi_{1}\right)\right)}\right)\times \left(\varphi_{1}+2\pi \times k_{1}\right)\right).$$

Here: exp() is the exponential function; j is the imaginary unit. Considering the frequency shift in such a signal:

(30) $$e^{j\left(a+b\right)\left(x+x_{0}\right)}\cdot e^{j\left(-a\right)x}=e^{j\left(\left(a+b-a\right)x+\left(a+b\right)x_{0}\right)}=e^{j\left(b\left(x+x_{0}\right)+ax_{0}\right)}.$$

Similar transformation is reasonable for expression (29): signal:

(31) $$\begin{array}{c} {\tilde{I}}_{PD2}^{c}\left(\varphi_{1}\right)=I_{PD2}^{c}\left(\varphi_{1}\right)\times \exp\left(j\times \left(-\frac{\Delta L_{21}}{\Delta L_{1}}\right)\right)\\ =\exp\left(j\times \left(\frac{L_{22}}{\Delta L_{1}}\times \frac{n_{2}\left(\omega \left(\varphi_{1}\right)\right)}{n_{1}\left(\omega \left(\varphi_{1}\right)\right)}\times \left(\varphi_{1}+2\pi \times k_{1}\right)+\frac{\Delta L_{21}}{\Delta L_{1}}\times 2\pi \times k_{1}\right)\right). \end{array}$$

The constant phase shift $\frac{\Delta L_{21}}{\Delta L_{1}}\times 2\pi \times k_{1}$ does not affect the amplitude-frequency characteristic, so it can be excluded from further consideration:

(32) $${\tilde{I}}_{PD2}^{c}\left(\varphi_{1}\right)=\exp\left(j\times \left(\frac{L_{22}}{\Delta L_{1}}\times \frac{n_{2}\left(\omega \left(\varphi_{1}\right)\right)}{n_{1}\left(\omega \left(\varphi_{1}\right)\right)}\times \left(\varphi_{1}+2\pi \times k_{1}\right)\right)\right).$$

Expression (32) is similar to (11), which describes a homogeneous reflectometer. Further signal processing will be carried out in a similar manner. First, the function Q(φ_1_, k_1_) should be introduced: consideration:

(33) $${\tilde{I}}_{PD2}^{c}\left(\varphi_{1}\right)=\exp\left(j\times \left(\frac{L_{22}}{\Delta L_{1}}\times \frac{n_{2}\left(\omega \left(\varphi_{1}\right)\right)}{n_{1}\left(\omega \left(\varphi_{1}\right)\right)}\times \left(\varphi_{1}+2\pi \times k_{1}\right)\right)\right)=\exp\left(j\times \left(\frac{L_{22}}{\Delta L_{1}}\times Q\left(\varphi_{1},k_{1}\right)\right)\right).$$

Then the phase ${\tilde{I}}_{PD2}^{c}\left(\varphi_{1}\right)$ of the signal is determined and unwrapped over the interval $\varphi_{1}\in \left[\varphi_{1}\left(0\right),\varphi_{1}\left(t_{end}\right)\right]$:

(34) $$\varphi_{2}\left(\varphi_{1}\right)+2\pi \times k_{2}=\frac{\Delta L_{22}}{\Delta L_{1}}\times Q\left(\varphi_{1},k_{1}\right).$$

After this, φ_2_(φ_1_) and Q(φ_1_, k_1_) are expanded into power series. Identical transformations are then performed and the expansion coefficients of Q(φ_1_, k_1_) are determined:

(35) $${\tilde{I}}_{PD2}^{c}\left(\varphi_{1}\right)=\exp\left(j\times \left(\frac{L_{22}}{\Delta L_{1}}\times q_{1}\times \left(1+{q^{\prime }}_{2}\times \varphi_{1}+{q^{\prime }}_{3}\times \varphi_{1}^{2}\right)\times \varphi_{1}\right)\right).$$

Let us introduce the equivalent phase function of the reference interferometer:

(36) $${\tilde{\varphi }}_{1}\left(\varphi_{1}\right)=\left(1+{q^{\prime }}_{2}\times \varphi_{1}+{q^{\prime }}_{3}\times \varphi_{1}^{2}\right)\times \varphi_{1}.$$

Then one can express the signal as a function of the equivalent phase:

(37) $${\tilde{I}}_{PD2}^{c}\left({\tilde{\varphi }}_{1}\right)=\exp\left(j\times \left(\frac{L_{22}}{\Delta L_{1}}\times q_{1}\times {\tilde{\varphi }}_{1}\right)\right).$$

As a result, the reflector’s signal amplitude-frequency response ${\tilde{I}}_{PD2}^{c}\left({\tilde{\varphi }}_{1}\right)$ exhibits a single narrow peak. The overall signal processing scheme is as follows:

(38) $$I_{PD1}\left(t\right)\to \varphi_{1}\left(t\right)\to I_{PD2}\left(\varphi_{1}\right)\to I_{PD2}^{c}\left(\varphi_{1}\right)\to {\tilde{I}}_{PD2}^{c}\left(\varphi_{1}\right)\to {\tilde{I}}_{PD2}^{c}\left({\tilde{\varphi }}_{1}\right).$$

First, the phase function of the reference interferometer signal over time φ_1_(t) is determined. Then, the signal of the second interferometer is expressed as a function I_PD2_(φ_1_) of the phase of the first interferometer. After this, the complex signal I^C^_PD2_(φ_1_) is calculated based on the real signal I_PD2_(φ_1_). The next processing step is to shift the frequencies ${\tilde{I}}_{PD2}^{c}\left(\varphi_{1}\right)$ of the received signal by an amount corresponding to the point where the radiation exits the fiber into the air. The final transformation involves a transition to the equivalent phase ${\tilde{I}}_{PD2}^{c}\left({\tilde{\varphi }}_{1}\right),$ which eliminates the influence of the inhomogeneity of the refractive indices of the medium.

## 4. Testing the Method of Eliminating Chromatic Dispersion

**Figure 3:**
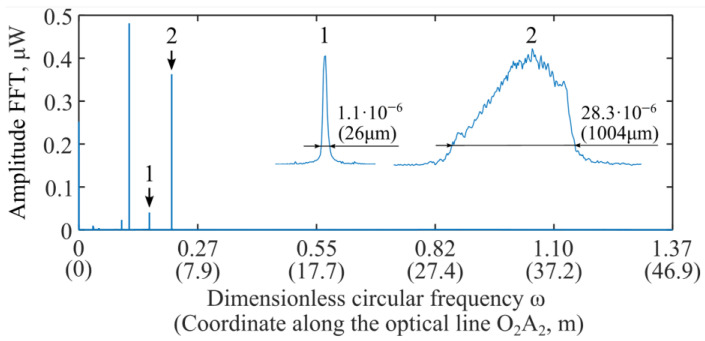
Experimentally obtained amplitude-frequency response of the signal I_PD2_(φ_1_). Peak widths are indicated at the −10 dB level, corresponding to the operating threshold of the center-of-mass detection algorithm.

The previous section described a method for eliminating the influence of refractive index inhomogeneities caused by different chromatic dispersions in quartz fiber and air. Further, it will be considered in relation to the experimental measurement results obtained on the setup shown in Figure 1. Figure 3 shows the amplitude-frequency response I_PD2_(φ_1_) of the signal for the case where the corner reflector is located at a distance of 1.8 m from the collimator (C_2_A_2_ = 1.8 m, see Figure 2). The graph shows two abscissa axes: the first is the dimensionless circular frequency ω, the second is the coordinate along the optical path O_2_A_2_. Here, a calibration coefficient, which will be determined in the next section, is used to calculate the coordinate.

The graph shows two peaks. The first corresponds to the optical radiation exit point from the collimator, and the second to the corner reflector. These peaks are shown in magnification in the free area of the graph. The scaling factors for the peaks vary. To quantify the peak widths, the corresponding dimensions are indicated. According to these, the width of the second peak is ≈38 times larger than the first. The broad peak obtained at the corner reflector is due to chromatic dispersion in the fiber and, to a much lesser extent, dispersion in air. This clearly illustrates the need for further signal processing, according to the general algorithm (30).

The next processing step is to determine the angular frequency ω_C_, which corresponds to the point C_2_ on the amplitude-frequency characteristic (see Figure 2, transition from fiber to air). The frequency value is determined by a previously developed algorithm [57]. This algorithm calculates the center of mass of the peak’s region above a −10 dB threshold relative to its maximum. It involves determining the center of mass of the peak’s apex. This algorithm improves the accuracy of frequency determination.

**Figure 4:**
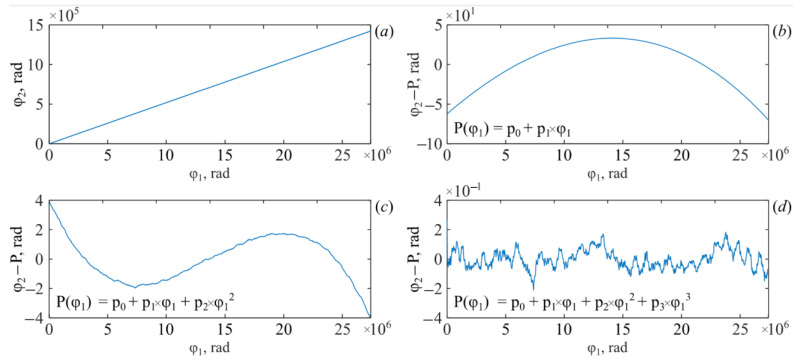
Expansion of the phase of the second interferometer into a power series. The phase of the resulting signal (**a**), phase of the resulting signal minus the linear component (**b**), The phase of the resulting signal minus the second-degree polynomial (**c**), The phase of the resulting signal minus the third-degree polynomial (**d**).

Then the signal spectrum is shifted by −ω_C_. The resulting signal is ${\tilde{I}}_{PD2}^{c}\left(\varphi_{1}\right)$. Its phase φ_2_φ_1_ is determined and expressed as a function of the phase of the first interferometer. The graph of this dependence is shown in Figure 4a. It is close to linear, but excluding the linear component (Figure 4b) clearly illustrates the presence of nonlinear terms. Describing the phase using a second-degree polynomial also contains a significant approximation error (Figure 4c). Only starting with a third-degree polynomial does the approximation describe the experimental dependence sufficiently well. This is demonstrated by Figure 4d.

The coefficients of a third-degree polynomial have the following values:

(39) p_0_ = 2.08, p_1_ = 4.29 × 10^−2^, p_2_ = −2.56 × 10^−13^, p_3_ = −2.20 × 10^−21^.

Based on these coefficients, the decomposition parameters Q(φ_1_, k_1_) are calculated:

(40) $${q^{\prime }}_{2}=\frac{p_{2}}{p_{1}}=5.96\times 10^{-12},\;\;\;{q^{\prime }}_{3}=\frac{p_{3}}{p_{1}}=5.13\times 10^{-20}.$$

**Figure 5:**
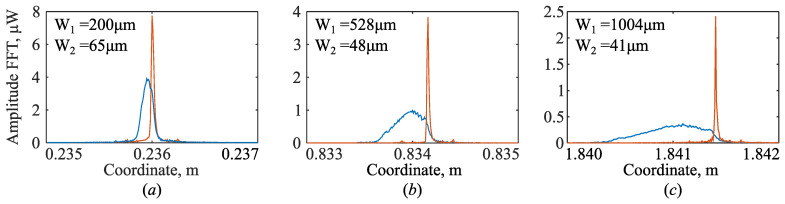
Three measurements were taken at different distances from the collimator: about 0.24 m (**a**), about 0.83 m (**b**), and about 1.84 m (**c**). Traces in the area of the corner reflector at various distances from the collimator. The original peak shape is shown in blue, and the orange one was obtained after correction for the refractive index function. The peak widths W_1_ (uncompensated) and W_2_ (compensated), indicated on the plot, are measured at the −10 dB level.

Using these parameters, the equivalent phase function ${\tilde{\varphi }}_{1}(\varphi_{1})$ is determined according to (36). The photodetector signal ${\tilde{I}}_{PD2}^{c}\left(\varphi_{1}\right)$ is then expressed as a function of this quantity. As a result, the signal takes the form of ${\tilde{I}}_{PD2}^{c}\left({\tilde{\varphi }}_{1}\right)$. The transformations performed eliminate the negative influence of chromatic dispersion on the peak shape. This is clearly shown in Figure 5. It shows traces with and without chromatic dispersion compensation. They are shown in orange and blue, respectively.

Traces in Figure 5 demonstrate changes in peak shape as the corner reflector is moved away from the collimator by 0.2 m, 0.8 m, and 1.8 m, respectively. It can be seen that with increasing distance, the peak obtained without chromatic dispersion compensation becomes spatially blurred, while after compensation, it remains constant in width. It should be noted that with increasing distance, the difference between the centers of the “compensated” and “uncompensated” peaks also increases. This means that the described procedure cannot be replaced by simply searching for the center of a spatially blurred peak without losing the accuracy of its localization.

## 5. Reflectometer Calibration

During the calibration process, measurements were performed in two modes. The first was an optical frequency domain reflectometry method, including chromatic dispersion compensation. The second was a phase-tracking fringe-counting method, which allows for highly accurate determination of reflector displacement. In reflectometer mode, measurements were performed in the typical OFDR manner: the light source was tuned from 1480 nm to 1640 nm, and its radiation was sent to two interferometers: the main and reference, as described above. The reference displacement was measured by a fringe-counting interferometric method. It was implemented using the same main interferometer (Figure 1b), but with the laser operating at a fixed wavelength of 1550 nm (without frequency sweep). In this configuration, a change in the optical path difference between the interferometer arms generates a harmonic interference signal (beats), where one full oscillation period corresponds to a displacement of λ/2 of the corner reflector. The recorded interference signal was processed to extract its phase, enabling displacement tracking with sub-wavelength accuracy. The calibration algorithm is described below.

According to (37), the circular frequency of the photodetector signal is given by:

(41) $$\omega =\frac{L_{22}}{\Delta L_{1}}\times q_{1}.$$

In this relationship, ω is known, and L_22_ is the desired value, which is equal to the distance between the collimator and the corner reflector. The quantities ΔL_1_ and q_1_ are constants whose values are known with low accuracy. To determine them more precisely, a calibration procedure is performed. It includes several measurement cycles. Each cycle consists of the following steps: first, the corner reflector is set to its initial position; then the trace is obtained, and based on it, the circular frequency ω_1_ corresponding to the reflector position is calculated; then the reflector is moved, and the magnitude of the movement is determined by the fringe counting method; after the move, the trace and the frequency value ω_2_ corresponding to the new reflector position are again determined; at the end of the measurement cycle, the reflector is returned to its initial position.

As a result of the measurement cycle, the following relationships can be written:

(42) $$\omega_{1}=\frac{q_{1}}{\Delta L_{1}}\times L_{22\_1},$$

(43) $$\omega_{2}=\frac{q_{1}}{\Delta L_{1}}\times L_{22\_2},$$

where L_22_1_ and L_22_2_ are the reflector’s coordinates before and after the movement. Subtracting (42) from (43):

(44) $$\omega_{2}-\omega_{1}=\frac{q_{1}}{\Delta L_{1}}\times \left(L_{22\_2}-L_{22\_1}\right).$$

The difference in the reflector’s coordinates is equal to the displacement measured by the fringe counting method. Therefore, expression (44) can be rewritten as follows:

(45) $$\Delta \omega =\frac{q_{1}}{\Delta L_{1}}\times \Delta X,$$

where ΔX is the reflector displacement measured by fringe counting; Δω is the change in angular frequency measured by reflectometry. During calibration, the constants q_1_ and ΔL_1_ cannot be determined separately, so the calibration parameter k should be introduced as follows:

(46) $$\Delta \omega =\frac{1}{k}\times \Delta X.$$

**Figure 6:**
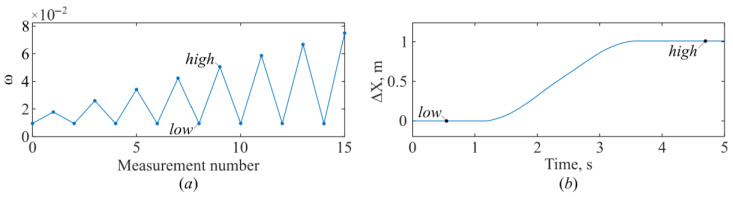
Measuring process. The system’s response variation with movement of the corner reflector (**a**), single movement of the corner reflector over time (**b**).

To accurately determine this, a series of cycles with varying displacement amplitudes is performed. The initial displacement amplitude is 0.2 m. With each cycle, it increases by 0.2 m. The measurement results are shown in Figure 6.

Figure 6a shows the angular frequency ω, corresponding to a series of measurement cycles. Figure 6b shows the displacement obtained using the fringe counting method, which corresponds to the 5th measurement cycle.

**Figure 7:**
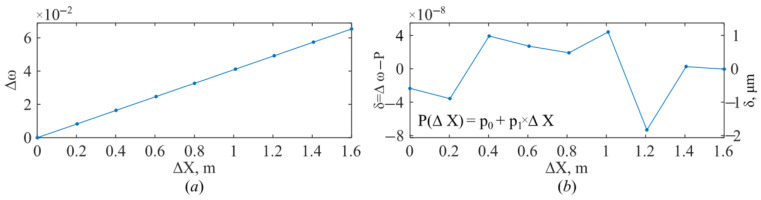
Reflectometer calibration process: the dependence Δω(ΔX) (**a**); the approximation error (**b**).

A dependence Δω(ΔX) was plotted for the series of measurements. It is shown in Figure 7a. A linear approximation of this dependence was performed using the least-squares method. The approximation error is shown in Figure 7b.

According to (46), the calibration parameter is expressed through the approximation parameter as follows:

(47) $$k=\frac{1}{p_{1}}.$$

As a result of the calibration, the coefficient k is determined with high accuracy. For our system, it is 24.56332 m. This constant physically encapsulates the ratio ΔL_1_/q_1_, thereby converting the dimensionless frequency scale of the processed signal into an absolute distance in meters for this specific experimental configuration. Using this coefficient, the distance between the collimator and the reflector is determined as follows:

(48) $$L_{22}=k\times \omega .$$

The maximum deviation of the circular frequency value from the linear approximation (Figure 7b) is 8 × 10^−8^. The calibration coefficient k allows one to determine the approximation error on a linear scale. It is 2 μm. Thus, the maximum error in determining the distance does not exceed 2 μm.

Thus, the measurement system is characterized by three parameters. Two of them are necessary to eliminate chromatic dispersion. The third parameter determines the relationship between circular frequency and distance.

### Discussion of Measurement Uncertainties

The achieved accuracy of 2 μm represents the maximum deviation observed during the calibration procedure. This value accounts for the residual uncertainties after applying the chromatic dispersion compensation. The key advantage of the proposed compensation is that it eliminates the distance-dependent systematic error caused by differential dispersion, which otherwise leads to peak broadening (Figure 5). The remaining error budget is attributed to the stability of the calibration parameter k, minor environmental fluctuations over the short air path, and fundamental noise sources (e.g., photon noise, ADC quantization). It is noteworthy that the center-of-mass peak detection algorithm used on the compensated trace allows for localization precision exceeding the nominal spatial resolution of the OFDR system. For applications requiring only absolute distance to a single reflector, phase-tracking interferometric methods might offer higher accuracy. However, the focus of this work is on extending the distributed sensing capability of OFDR to heterogeneous lines, which necessitates the spectral analysis performed.

## 6. Conclusions

In this study, the optical frequency domain reflectometry method was adapted for high-precision length gauging in air. This study demonstrates that, although OFDR is traditionally used to measure optical lengths to backscattering points in optical fibers and integrated optical chips, its potential for measuring absolute distances in open space remains poorly understood.

The main challenge in using the OFDR method for measuring distances in air is analyzing optical lines with heterogeneous compositions. Part of the line may be traditionally represented by optical fiber, while the rest of the line is radiated in free space. In this case, light propagates through media with significantly different refractive indices. The situation is further complicated by the fact that the chromatic dispersion functions for optical fiber and air are different. As a result, the peak in the trace broadens with increasing distance from the reflector to the point where the light exits the fiber. This significantly reduces the accuracy of reflector position determination. This paper proposes a method that eliminates the negative impact of chromatic dispersion on measurement results.

It is worth noting that a similar situation can also occur in classical optical frequency domain reflectometry applications. When the reference and primary interferometers are made of different fiber types, a single point along the optical path corresponds to a diffuse peak in the trace. In this case, the accuracy of strain or temperature determination decreases as the distance to the measured point increases. The developed algorithm for eliminating the influence of chromatic dispersion can also be applied in this case.

Accurate distance measurement requires precise determination of the measurement system parameters. Typically, the lengths of the interferometer arms within a reflectometer, as well as the refractive indices of the media through which the radiation propagates, are not accurately known. Therefore, calibration is necessary for each reflectometer. This paper demonstrates that accurate distance determination using an OFDR requires the determination of one parameter. This parameter can be obtained by comparing the reflectometer readings with the interferometric displacement measurement method. As a result of calibration, a measurement system parameter was determined that ensures a distance measurement accuracy of 2 μm.

It can be concluded that an adapted version of OFDR is capable of providing accurate distance measurements in open space, opening up new horizons for the technology’s use in ranging and monitoring optical circuits with structures that contain materials with different optical properties. This is possible because the general methodology applied to an optical line consisting of just two parts with different optical properties can be extrapolated to more complex lines, where the refractive index and chromatic dispersion properties vary along the length according to a more complex law.

Since OFDR allows for the reconstruction of all points along an optical line, including those before and after a reflecting object, simultaneous profilometric measurements are possible in addition to range measurements. These measurements will allow not only the precise distance to an object but also its surface characterization.

In the described data processing algorithm, the signal from the primary interferometer is expressed as a function of the phase of the reference interferometer. Consequently, the resulting trace is independent of the speed and uniformity of the optical radiation frequency tuning. This significantly reduces the requirements for the laser radiation source.

Nevertheless, many challenges remain to be addressed for the widespread adoption of OFDR in various fields of science and technology, the most important being the development of inexpensive laser sources capable of linear frequency tuning. In this regard, the use of self-scanning fiber lasers, which are being developed by the Lobach et al. team [58,59,60], is of interest.

The use of such radiation sources will eliminate the need for auxiliary interferometers, as the radiation frequency sweeps strictly linear, and will significantly reduce the cost of the overall system. This type of source, emitting in the ytterbium luminescence band, has not yet found widespread use in the telecommunications industry because this wavelength does not coincide with the transparency window of germanium-doped quartz glass. This is less important for measuring lengths in air, so it is reasonable to believe that optical rangefinders based on fiber lasers offer significant potential for development and comprehensive commercialization.

Also, as further promising areas of research, one can highlight the testing of optical ranging methods in the time domain, similar in principle to phase-sensitive optical time domain reflectometry [61], as well as the integration of artificial intelligence methods into the signal processing chain [62].

## Figures and Tables

**Table 1:** The main components of the experimental setup.

Setup Element(s)	Brand/Manufacturer
Wavelength-tunable laser source	TSL-570-P-480640-P-F-AP-00-1 (Santec Holdings corp., Komaki, Aichi, Japan)
Infrared detectors	HCA-S-200M (FEMTO Messtechnik GmbH, Berlin, Germany)
Faraday mirrors	OFM-15-L-1-2 (AFW Technologies Pty Ltd., Hallam, Victoria, Australia)
Data acquisition board (analog-to-digital converter)	PCIE-1840L (Advantech Co., Ltd., Taipei, Taiwan, China)
Couplers and circulators	Advanced Fiber Resources, Ltd., Zhuhai, China

## Data Availability

The data reported in this manuscript are available on request from the corresponding author.

## Abbreviations

The following abbreviations are used in this manuscript:

OFDR	Optical frequency domain reflectometry
FEM	Finite element method
COPV	Composite pressure vessels
